# Interaction Between Aging-Related Elastin-Derived Peptide (VGVAPG) and Sirtuin 2 and its Impact on Functions of Human Neuron Cells in an In Vitro Model

**DOI:** 10.1007/s12035-024-04298-y

**Published:** 2024-06-24

**Authors:** Bartosz Skóra, Tomasz Piechowiak, Konrad A. Szychowski

**Affiliations:** 1 Department of Biotechnology and Cell Biology, Medical College, University of Information Technology and Management in Rzeszow, St. Sucharskiego 2, 35-225 Rzeszów, Poland; 2 Department of Chemistry and Food Toxicology, Institute of Food Technology and Nutrition, University of Rzeszow, St. Ćwiklinskiej 2, 35-601 Rzeszów, Poland

**Keywords:** VGVAPG, Sirtuin 2, Neurodegeneration, Neurons, SH-SY5Y, Elastin-derived-peptide

## Abstract

Elastin is a stable protein present in many tissues, including brain tissues, and is one of the most long-life proteins with a half-life of approximately 70 years. The peptide with a Val-Gly-Val-Ala-Pro-Gly (VGVAPG) amino acid sequence is released during elastin decay, which correlates with aging-related neurodegeneration. A recent study has shown enhanced protein expression of Sirtuin 2 (SIRT2 – one of the *redox* homeostatic factors) in aged rodent brains, while the correlation between VGVAPG and SIRT2 has never been evaluated so far. Therefore, the study aimed to determine the impact of the VGVAPG hexapeptide on SIRT2 and neuronal functions in differentiated SH-SY5Y cells at the gene and protein expression levels. The present results showed that VGVAPG caused a 52.69% decrease in the level of reactive oxygen species (ROS), as in the case of neurons treated with AGK2 (Sirtuin 2 inhibitor) after 24h and 48h. Furthermore, a decrease in superoxide dismutase (SOD) activity was observed. The *SIRT2* gene expression was found to fluctuate after 6h and 24h as a result of the exposure to the VGVAPG peptide. In turn, a decrease in the *PPARγ, P53, SOD2*, and *CAT* mRNA expression was shown in VGVAPG-treated cells. Additionally, an increase in the Sirtuin 2 protein expression was recorded after 24h and 48h in the VGVAPG peptide-treated neurons. Last but not least, the decrease in the level of acetylation of α-tubulin after the hexapeptide treatment was correlated with shortening of neurites, which may indicate the destabilization of the microtubule and ROS-independent induction of neurodegeneration.

**Graphical Abstract:**

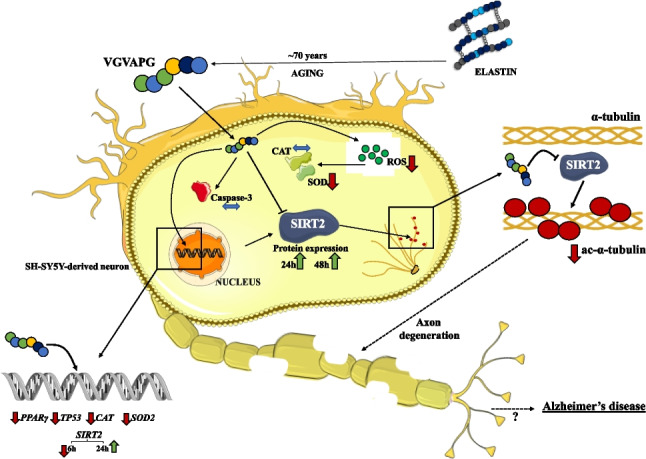

**Supplementary Information:**

The online version contains supplementary material available at 10.1007/s12035-024-04298-y.

## Introduction

Elastin is the main building-block of the extracellular matrix (ECM). This protein can be found e.g. in lungs, veins, and brains, where it is responsible for *inter alia* protection against mechanical damage by providing elasticity/flexibility to tissues [1]. As shown *inter alia* by Le Page et al. in their review, elastin is a low-turnover-time protein due to its long half-life, estimated at approximately 70 years, which was proved in 1981 by Shapiro et al. in *post-mortem* lung specimens [2, 3]. The physiological and/or pathological degradation of elastin results in release of so-called elastin-derived-peptides (EDPs), which are detected in many tissues, e.g. in infarction-affected brain (measured in cerebrospinal fluid and varying between 6.3 ng/mL to 129.5 ng/mL) and/or in human serum (depending on the tested group, the amount of EDPs varied between 11.9 ng/mL to 81.1 ng/mL) [4, 5]. In turn, the Val-Gly-Val-Ala-Pro-Gly (VGVAPG) peptide is considered to be the most easily liberated fraction from elastin, formed during the degradation of elastin [3, 6]. Moreover, as reviewed in previous studies and reported in the literature, the elastin degradation process may be accelerated (measured as an increase in the EDPs level) during stroke and as a result of overproduction of reactive oxygen species (ROS) [7, 8]. Recent studies show that this hexapeptide is able to affect the peroxisome proliferator-activated receptor gamma (Pparγ) in mouse astrocytes and undifferentiated neuroblastoma cells (SH-SY5Y) in vitro [9, 10]. Moreover, as reported by Jiang et al., the amount of this receptor is increased in the brains of AD patients [11], and Carta suggested that this receptor may be a promising target in Parkinson’s disease (PD) [12]. Furthermore, Ma et al. proved that elastin-like polypeptides (ELPs) are able to increase the amounts of β-amyloid (Aβ) isoforms (Aβ_40_ and Aβ_42_) in both in vitro and in vivo experiments [13]. However, due to the different structures of ELPs, the effect of the VGVAPG peptide (EDPs) in neurons cannot be predicted. Nevertheless, these results suggest that there is a major gap in the knowledge of the impact of the VGVAPG hexapeptide on the metabolism of neuronal cells, which may be a key factor in understanding the basis of neurodegeneration, especially given the recent study conducted by Ma et al. (2024), who showed that high (non-physiological) concentrations of VGVAPG are able to disturb the functioning of mouse hippocampal neuronal cells (HT-22) and activate mouse microglia cells (BV2) [14]. However, no studies have been performed using human-origin cells.

The NAD^+^-dependent deacetylase Sirtuin 2 (SIRT2) is present in many tissues, also in neurons, where it is responsible *inter alia* for microtubule stabilization through deacetylation of α-tubulin and/or is involved in the maintenance of intracellular *redox* homeostasis [15, 16]. Interestingly, a recent postmortem study (2021) of early-stage AD patients has shown that the brainstem is affected by AD symptoms in the earliest phase of the disease [17]. In turn, Sidorova-Darmos et al. showed that, compared to other sirtuins, the *Sirt2* mRNA expression (coding for the Sirtuin 2 protein) was up to threefold higher in aged rats. Moreover, the highest *Sirt2* gene expression was found in the brainstem and spinal cord [18]. The same correlation was proved at the protein level, showing an increase in the Sirt2 level during aging in rats [18]. Although the role of Sirtuin 2 in normal cell damage is confusing, this aging-related increase in the SIRT2 expression may correlate with the occurrence of neurodegeneration in the aged population. Moreover, it seems to be associated with the half-life of elastin degradation and the increasing amount of VGVAPG during aging and in injured brains (e.g. ischemic stroke), but this aspect has never been investigated before in any human-origin neuronal cell models. In this study, differentiated neuronal human neuroblastoma cells (SH-SY5Y) were used. According to the literature, such cells are a well-established model of human mature neurons, which makes them a proper model for screening studies [19, 20]. Furthermore, the fully-differentiated SH-SY5Y cells are characterized by morphological changes and increased levels of molecular markers characteristic for mature neurons [e.g. microtubule-associated protein 2 (MAP2), tubulin beta 3 class III (TUBB3)], in contrast to rodent-derived neuronal cultures, which are characterized by a lack of many isoforms of such proteins and differences in the tau sorting mechanisms [19]. What is more, such a phenotype appears only after the end of differentiation, hence these neuronal cells were chosen as a model in this study.

Therefore, the aim of the present study was to determine the impact of the VGVAPG hexapeptide and its interaction with Sirtuin 2 on neuronal function in differentiated SH-SY5Y cells as a model of neurons in vitro. The influence of this relationship on the basic metabolic parameters, neurite degradation, and mRNA and protein expression of certain genes/proteins was studied to reveal the role of VGVAPG-SIRT2 interactions in the potential development of neurodegeneration.

## Material and Methods

### Reagents

Trypsin, penicillin, streptomycin, resazurin sodium salt, 2',7'-dichlorodihydrofluorescein diacetate (H_2_DCF-DA), caspase-3 substrate (Ac-DEVD-pNA), hydroxyethyl piperazineethanesulfonic acid (HEPES), sodium chloride, 3-[(3-cholamidopropyl)dimethylammonio]-1-propanesulfonate hydrate (CHAPS), ethylenediaminetetraacetic acid (EDTA), glycerol, dithiothreitol (DTT), protease inhibitor cocktail, Bradford reagent, bovine serum albumin (BSA), Tween-20, alcohol dehydrogenase, epinephrine, ammonium metavanadate, hydrogen peroxide (H_2_O_2_), methanol, all-trans retinoic acid (RA), and acrylamide/bisacrylamide were purchased from MERCK (Darmstadt, Germany). The DMEM/F12 and phosphate buffer saline (PBS) were purchased from Corning (New York, USA). The fetal bovine serum heat inactivated (FBS HI), radioimmunoprecipitation assay (RIPA) buffer, Universal RNA Purification Kit, Perfect Tricolor Protein Ladder and Fast Probe qPCR Master Mix (2x), and plus ROX Solution were purchased from EURx (Gdańsk, Poland). The anti-GAPDH (sc-47724), anti-ac-α-tubulin (sc-23950) mouse primary antibodies and AGK2 (Sirtuin 2 inhibitor) were purchased from SantaCruz Biotechnology (Santa Cruz, USA). The goat anti-rabbit (#31460) and goat anti-mouse (#31430) secondary antibodies HRP-conjugated, TaqMan probes and starters complementary to genes encoding *GAPDH* (Hs02758991_g1), *TUBB3* (Hs00801390_s1), *SIRT2* (Hs01560289_m1), *P53* (Hs01034249_m1), *CAT* (Hs00156308_m1), and *SOD2* (Hs00167309_m1), and the High-Capacity cDNA Reverse Transcription Kit were purchased from Thermo Fisher Scientific (Waltham, USA). The Laemmli Sample Buffer and β-mercaptoethanol (BME) were purchased from Bio-Rad (Hercules, USA). The rabbit anti-SIRT2 primary antibodies (A3967) were purchased from ABClonal (Woburn, USA). The mouse anti-α-tubulin primary antibodies (66031–1-Ig) were purchased from Proteintech (Düsseldorf, Germany).

### Cell Culture and Differentiation Protocol

The human neuroblastoma cell line (SH-SY5Y, ATCC® CRL-2266) was purchased from the American Type Culture Collection (ATCC). The cells were cultured in DMEM/F12 with 10% of FBS HI, supplemented with 0.1% of penicillin/streptomycin, in a humidified atmosphere (37°C, 5% CO_2_). After reaching confluency, the cells were seeded on a 96-well plate at the density of 4.5 × 10^3^ cells/well, on a 12-well plate at the density of 9 × 10^4^ cells/well, on a 6-well plate at the density of 12 × 10^5^ cell/well, or on a ⌀35 mm culture dish at the density of 1 × 10^5^ cells/well 24 h before the experiment. After this time, the differentiation was initiated as in Zhang et al. with the use of a differentiation medium containing DMEM/F12, 1% of FBS HI, and 10 µM of all-trans retinoic acid (RA) [21]. The medium was subsequently replaced every 3 culture days up to the 14^th^ day to obtain mature neuron model. The effectiveness of the differentiation was assessed based on *TUBB3* mRNA expression (a well-established marker of neurons) and visualized using fluorescence staining. Next, on the 14^th^ day of differentiation (mature neurons), the differentiation medium was removed and replaced with medium containing 10 µM of AGK2 and 10 nM of VGVAPG. The concentrations of the tested compounds were chosen based on a previous study [9] and literature data [22]. Additionally, to determine the effect of the tested peptide on Sirtuin 2, the cells were first pretreated with 10 µM of AGK2 (for 0.5 h); afterwards, the cells were treated with 10 nM of VGVAPG for 24h or 48h,. The differentiated neuronal SH-SY5Y cells were used as a model of mature neurons in all the experiments.

### Resazurin Reduction Assay

The resazurin reduction assay was performed as in Szychowski et al. [9]. Briefly, on the 14^th^ day of differentiation, the medium was removed and replaced with medium containing 10 µM of AGK2 or 10 nM of VGVAPG. Additionally, pretreatment with 10 µM of AGK2 was performed for 0.5h before the treatment of the SH-SY5Y cells with 10 nM of VGVAPG. After 24h or 48h, the medium was removed and replaced with serum-free medium containing 1% resazurin sodium salt. The measurement of fluorescence was performed at λ_ex._ = 530 nm and λ_em._ = 590 nm using a microplate reader (FilterMax F5). The results were expressed as a percentage (%) of the control (DMSO-treated cells).

### Intracellular Reactive Oxygen Species (ROS) Level

The 2',7'–dichlorofluorescin diacetate (H_2_DCF-DA) assay was performed as proposed by Skóra et al. [23]. Briefly, on the 14^th^ day of differentiation, the medium was replaced with serum-free medium containing 5 µM of H_2_DCF-DA and incubated for 30 min at 37°C with 5% CO_2_. After this time, the cells were washed once with warm PBS to remove probe residues, and fresh medium containing 10 µM of AGK2 or 10 nM of VGVAPG was added. Additionally, pretreatment with 10 µM of the AGK2 was performed for 0.5h before the treatment of the neuronal cells with 10 nM of VGVAPG. After 24h and 48h, the measurement of fluorescence was performed at λ_ex._ = 485 nm and λ_em._ = 535 nm using a microplate reader (FilterMax F5). The results were expressed as a percentage (%) of the control (DMSO-treated cells).

### Caspase-3 Activity Assay

The caspase-3 activity assay was performed as in Nicholson et al. with minor modifications [24]. Briefly, after 24h or 48h of treatment of the cells with the tested compounds, the cells were frozen at -80°C for 24h. Afterwards, the cells were lysed using CAB buffer (50 mM HEPES, pH 7.4, 100 mM NaCl, 0.1% CHAPS, 1 mM EDTA, 10% glycerol, 10 mM DTT) for 10 min at 4°C. Subsequently, the caspase-3-specific substrate (Ac-DEVD-pNA) was added for 30 min. Next, absorbance was measured using a microplate reader (FilerMax F5) at λ = 405 nm. The results were expressed as a percentage (%) of the control (DMSO-treated cells).

### Confocal Fluorescence Microscopy

Fluorescence confocal microscopy was used in this study to determine the impact of the tested compounds on degeneration of axons, which is a well-described indicator of the phenotype of AD-like neurons. This method was used to confirm the effectiveness of the neuron differentiation procedure. Briefly, on the 14th day of differentiation, the cells were treated with the tested compounds for 72h as described above. Next, the medium was removed and the cells were washed 3 times with warm PBS and stained with serum-free medium containing 10 µM of Hoechst 33342 (cell nucleus dye) and 10 µM of Calcein-AM (cell cytoplasm dye) for 5 min (37°C, 5% CO_2_). Next, the cells were visualized by confocal microscopy with a laser scanner module at × 100 magnification (ZEISS LSM700). The mean axon length was quantified using the ImageJ and AutoNeuriteJ module as proposed by Boulan et al. [25]. The results were expressed as a percentage (%) of the control (DMSO-treated cells).

### Superoxide Dismutase and Catalase Activity

Superoxide dismutase (SOD) and catalase (CAT) activities were used in this study as markers of oxidative stress in the cells. After treatment with the tested compounds on the 14^th^ day of differentiation, the cells were collected using 50 mM TRIS (pH = 7.2) and frozen at -80°C. Subsequently, the SOD and CAT activity measurement was performed as described by Piechowiak et al. with modifications introduced by Szychowski et al. [23, 26]. The absorbance was measured at λ = 490 nm and λ = 374 nm for SOD and CAT activity, respectively, using a microplate reader (EPOCH). The results were expressed as a percentage (%) of the control (DMSO-treated cells).

### Real-Time Polymerase Chain Reaction (RT-PCR)

The RT-PCR assay was performed as in Szychowski et al. with minor modifications [27]. Briefly, after 6h or 24h (in the case of SIRT2) of treatment of the neurons with the tested compounds, the total mRNA was obtained using the Universal RNA Purification Kit according to the manufacturer’s manual (EURx). Subsequently, the reverse transcription reaction was performed using the High-Capacity cDNA Reverse Transcription Kit (ThermoFisher). Next, the obtained cDNA template was used in the RT-PCR reaction mixture (total volume 20 µL) containing Fast Probe qPCR Master Mix (2x), primers, and TaqMan probes specific for genes encoding *GAPDH, TUBB3, SIRT2, PPARγ, CAT*, and *SOD2*. The RT-PCR method used in this study consisted of the following steps: 2 min at 50 °C and 10 min at 95 °C, followed by 45 cycles of 15 s at 95 °C and 1 min at 60 °C. The threshold value (Ct) for each sample was calculated during the exponential phase and ΔΔ_Ct_ was used to determine the Average Fold (Avg. Fold) of the expression of certain genes. *GAPDH* was used as a reference gene.

### Western Blot Analysis

The protein expression was performed as in Szychowski et al. with minor modifications [28]. Briefly, after 24h and 48h of treatment, the differentiated SH-SY5Y cells were lysed using RIPA buffer containing protease inhibitor cocktail and sonicated (amp. 10%, 8s.) on ice. Subsequently, the total protein content in each sample was measured using the Bradford method (BSA as a standard), and the concentration of protein in each sample was standardized. Next, 40 µg of each sample was mixed with 5 × Laemmli buffer and the samples were separated in 7.5% SDS–polyacrylamide gel electrophoresis (Bio-Rad). Afterwards, the proteins were transferred onto the PVDF membrane and left overnight at 4°C with 35V. Subsequently, non-specific side blocking was performed using 1% BSA in TBST buffer for 1 h, followed by overnight incubation of the membranes with anti-GAPDH (1:1500), anti-SIRT2 (1:2000), and anti-Ac-α-Tubulin (1:400) primary antibodies at 4°C. After this time, the membranes were washed 3 times with TBST and horseradish peroxidase-conjugated anti-mouse or anti-rabbit secondary antibodies (1:10 000 for anti-Sirtuin 2 and Ac.-α-tubulin, 1:20 000 for anti-GAPDH) were added for 1h at RT. Next, the membranes were washed 3 times with TBST buffer and once with 1 × TBS for 10 min. The protein amount was measured using the chemiluminescence method induced by luminol reagent (Li-Cor). The results were expressed as a relative protein level normalized to GAPDH as a reference protein in each sample and compared to the control (DMSO-treated cells).

### Statistical Analysis

In this study, the data are presented as mean values ± standard deviations (SD). Each experiment was performed at least 3 times (n ≥ 3). The results were subsequently subjected to statistical analysis performed using the GraphPad Prism 8.0 Statistical Analysis Mode. The analysis of the statistical differences between the control and treated cells was performed using one-way ANOVA with Tukey’s post-hoc test. The statistical difference between certain groups was measured using the t-test. The results of the statistical analyses (*p* values) are presented under the bars in each graph.

## Results

### Metabolic Activity and Intracellular ROS Level

**Fig. 1:**
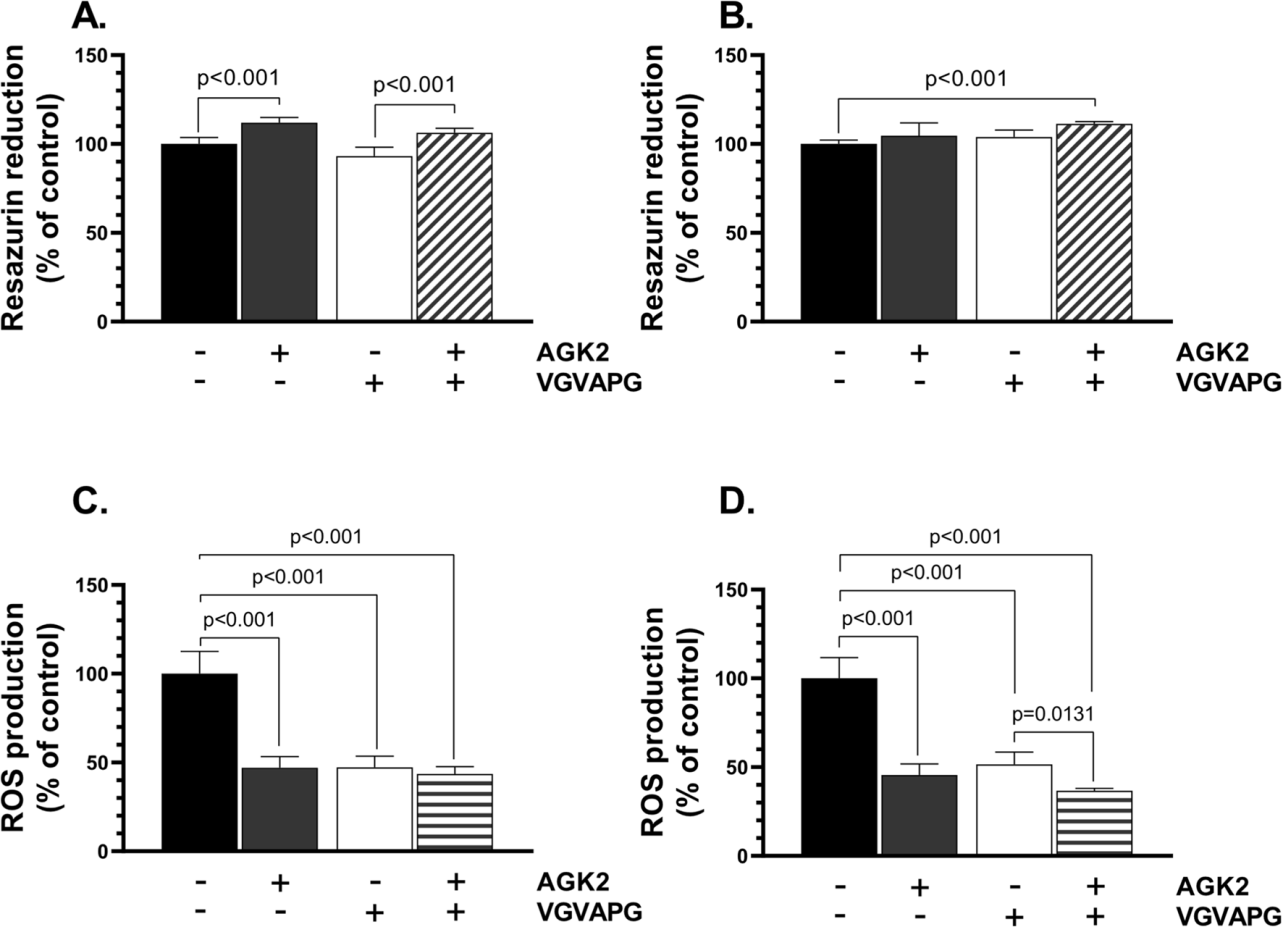
The VGVAPG hexapeptide does not impact the metabolic activity but significantly decreases the intracellular ROS level in the tested neuronal cell model. Results of the metabolic activity (**A**-**B**) and intracellular ROS level (**C**-**D**) in SH-SY5Y-derived neurons after treatment with 10 µM of AGK2, 10 nM of VGVAPG, and 10 µM of AGK2/10 nM of VGVAPG after 24 h (A, C) or 48 h (B, D). The data are presented as means ± SD. The p values of one-way ANOVA, compared to the control, as well as p values shown by the t-test analysis between certain groups are denoted under the bars. The black, dark grey, white, and patterned bars show the results after the treatment with the vehicle (Control), VGVAPG, AGK2, and VGVAPG/AGK2, respectively

The resazurin reduction assay was chosen to determine the impact of the tested hexapeptide and the SIRT2 inhibitor on metabolic activity in the chosen cell model. The 24-h treatment of the differentiated SH-SY5Y cells with AGK2 caused an 11.94% increase in the metabolic activity, compared to the control, while no changes in this parameter were observed in the treatments with the other compounds, compared to the control (Fig. 1A). The effects of VGVAPG and AGK2/VGVAPG on the analyzed cells were statistically different (Fig. 1A). After the 48-h treatment, no significant changes in the metabolic activity were induced by any of the tested compounds; however, an 11.26% increase in this parameter was exhibited by the AGK2/VGVAPG-treated cells, compared to the control (Fig. 1B).

The H_2_DCF-DA probe is a membrane-permeable probe, which is initially modified by intracellular esterases, followed by a reaction with intracellular ROS. Therefore, this method was chosen in this study to determine the impact of VGVAPG and the SIRT2 inhibitor on the intracellular ROS level in the neurons. Compared to the control, the intracellular ROS level in the AGK2-, VGVAPG-, and AGK2/VGVAPG-treated cells decreased after the 24-h treatment by 52.99%, 52.68%, and 56.47%, respectively (Fig. 1C). Similar results were obtained after the 48-h treatment of the neuronal cells with the tested compounds, which reduced the ROS level by 44.48%, 48.46%, and 53.30% in the AGK2-, VGVAPG-, and AGK2/VGVAPG-treated cells, respectively, compared to the control (Fig. 1D). Additionally, statistically different ROS levels between the VGVAPG- and AGK2/VGVAPG-treated cells were observed (Fig. 1D).

### Caspase-3 Activity

**Fig. 2:**
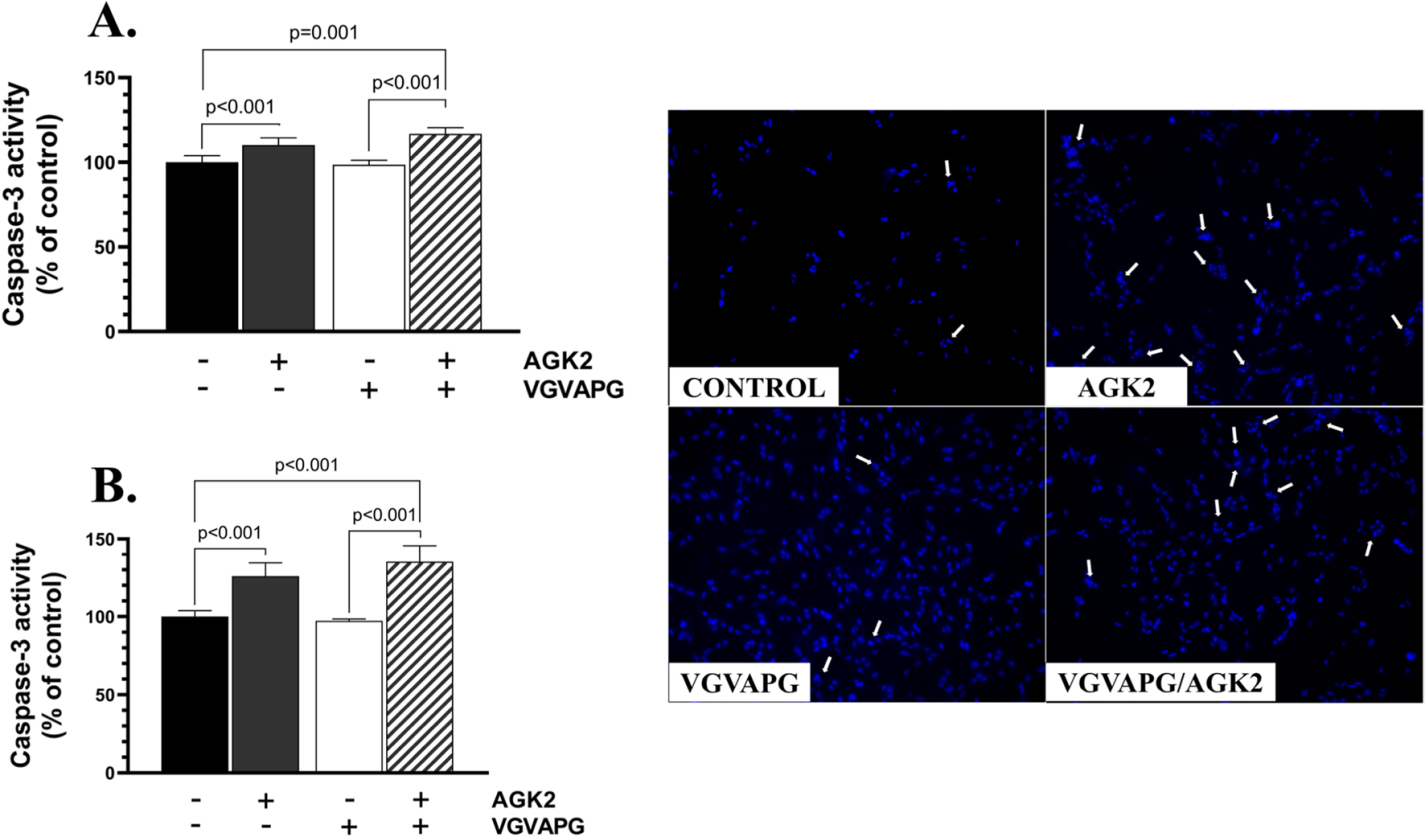
The tested hexapeptide does not induce active apoptosis in the tested neuronal cell model. Results of caspase-3 activity in SH-SY5Y-derived neurons after 24 h (**A**) and 48 h (**B**) of treatment with AGK2, VGVAPG, and VGVAPG/AGK2 and representative images of Hoechst33342-stained cells (right panel). The white arrows indicate apoptotic nuclei. The data are presented as means ± SD. The p values of one-way ANOVA, compared to the control, as well as p values shown by the t-test analysis between certain groups are denoted under the bars. The black, dark grey, white, and patterned bars show the results after the treatment with the vehicle (Control), VGVAPG, AGK2, and VGVAPG/AGK2, respectively

Caspase-3 is an effector caspase and a well-described marker of active apoptosis [29]. In this study, the level of activity of this protein was chosen as an indicator of the pro-apoptotic ability of the tested hexapeptide and the SIRT2 inhibitor. Caspase-3 activity increased by 10.20% in the AGK2-treated cells, compared to the control, while no such effect was observed in the VGVAPG-treated cells after 24h (Fig. 2A). The differentiated SH-SY5Y cells treated with AGK2/VGVAPG showed a 16.75% increase in caspase-3 activity, compared to the control (Fig. 2A). After the 48-h treatment, the cells treated with AGK2 exhibited a 20.05% increase in caspase-3 activity, compared to the control (Fig. 2B). The VGVAPG/AGK2-treated cells showed a 35.17% increase in caspase-3 activity, compared to the control; moreover, this effect was statistically different (by 37.86%) from that in the VGVAPG-treated cells (Fig. 2B).

### Axon Degeneration

**Fig. 3:**
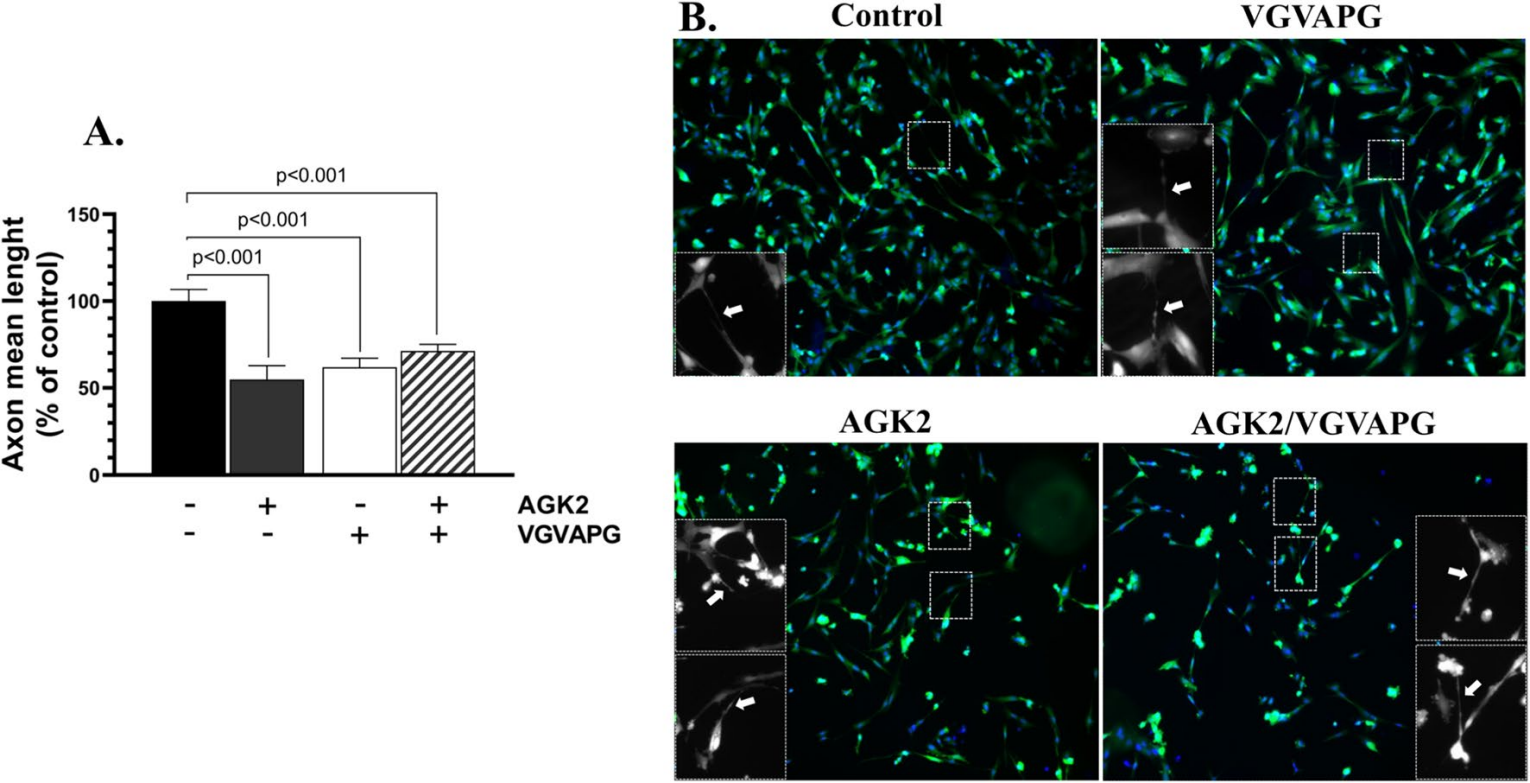
The tested VGVAPG causes shortening of the neurite length in the tested neuronal cell model. Mean axon length in SH-SY5Y-differentiated neurons after 72-h treatment with VGVAPG, AGK2, and VGVAPG/AGK2 quantified by AutoNeuriteJ ImageJ plugin (**A**) and representative images with an inset showing the degeneration process of the axon (white arrows) (**B**). The data are presented as means ± SD. The p values of one-way ANOVA, compared to the control, as well as p values shown by the t-test analysis between certain groups are denoted under the bars. The black, dark grey, white, and patterned bars show the results after the treatment with the vehicle (Control), VGVAPG, AGK2, and VGVAPG/AGK2, respectively

The shortening of axon length is one of the characteristic features of neurodegenerative cells [30]. Therefore, this type of morphology-based measurement was chosen to determine the ability of the tested hexapeptide and/or the SIRT2 inhibitor to induce changes in this parameter. AGK2 caused a 45.18% decrease in the mean axon length, compared to the control, and a similar effect was observed in the VGVAPG-treated cells, which were characterized by a 38.01% decrease in this parameter, compared to the control (Fig. 3A). These morphological changes in the axons were observed in both groups (Fig. 3B). The AGK2- and VGVAPG-treated cells also exhibited a 28.78% decrease in the axon length, compared to the control (Fig. 3A).

### CAT and SOD Activity

**Fig. 4:**
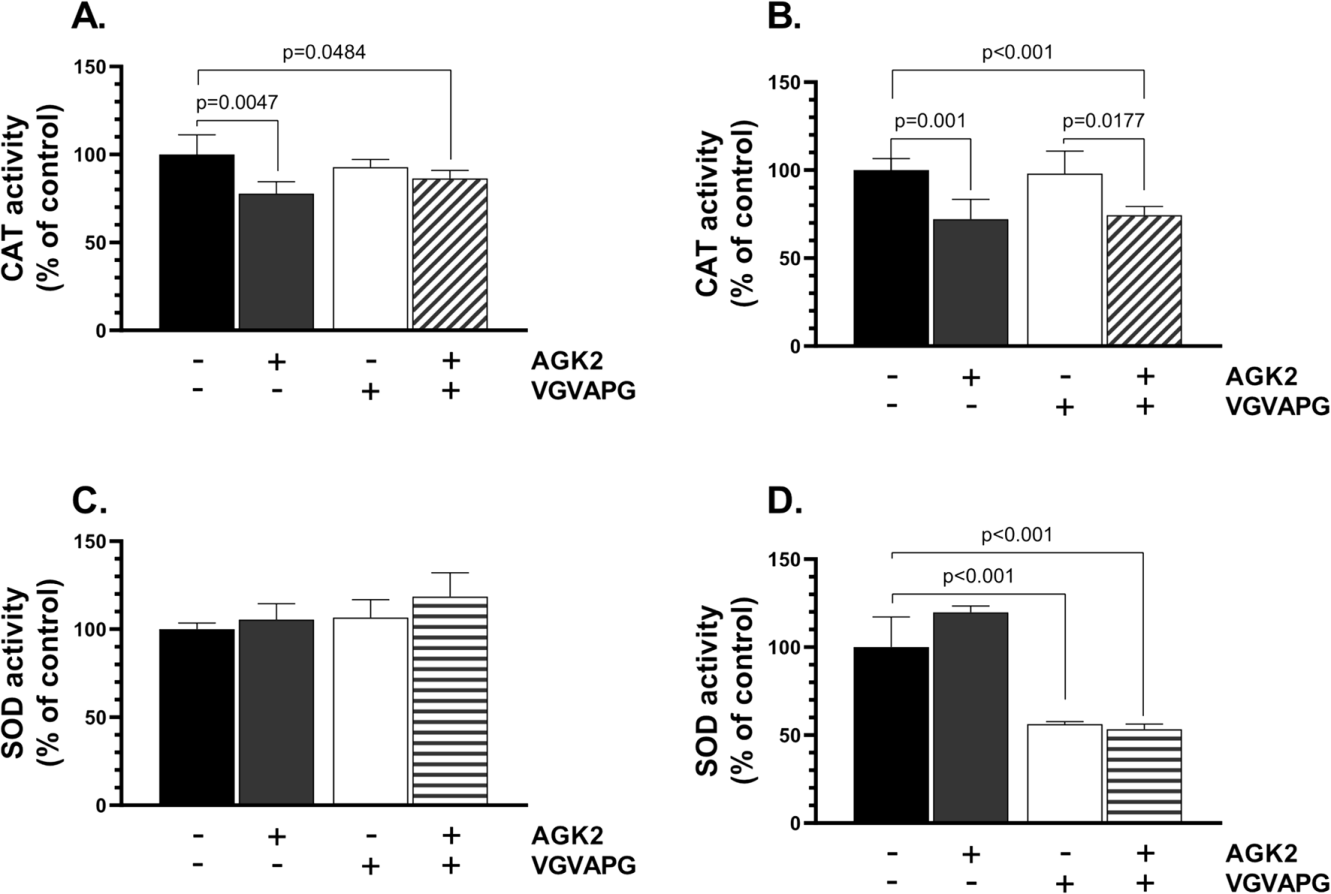
The VGVAPG does not induce oxidative stress in the neuronal cell model. Results of CAT (**A**-**B**) and SOD (**C**-**D**) activity after treatment of the SH-SY5Y-derived neurons with AGK2, VGVAPG, and VGVAPG/AGK2 for 24 h (A-C) and 48 h (B-D). The data are presented as means ± SD. The p values of one-way ANOVA, compared to the control, as well as p values shown by the t-test analysis between certain groups are denoted under the bars. The black, dark grey, white, and patterned bars show the results after the treatment with the vehicle (Control), VGVAPG, AGK2, and VGVAPG/AGK2, respectively

CAT and SOD are two main enzymes involved in the intracellular antioxidant system in cells. An increase in their activity is usually correlated with the occurrence of oxidative stress [31]. Accordingly, we have chosen this method to measure the pro-oxidative ability of VGVAPG and the SIRT2 inhibitor in the tested in vitro neuron model. The 24-h treatment of the differentiated SH-SY5Y cells with AGK2 reduced CAT activity by 22.17%, compared to the control (Fig. 4A). The VGVAPG/AGK2-treated cells showed a 13.51% decrease in this parameter, compared to the control (Fig. 4A). In turn, no changes in CAT activity were observed in the VGVAPG-treated cells (Fig. 4A). After 48h, the SH-SY5Y cells exhibited a 27.79% and 25.55% decrease in CAT activity in the AGK2 and VGVAPG/AGK2 treatment variants, respectively, compared to the control (Fig. 4B). No changes in this parameter were observed in the VGVAPG alone-treated cells (Fig. 4B).

After 24h, no statistically significant changes in SOD activity were induced by the treatments with the tested compound (Fig. 4C). In turn, a 43.72% and 46.63% decrease in the SOD activity was observed after 48h in cells treated with VGVAPG and VGVAPG/AGK2, respectively, compared to the control (Fig. 4D).

### *TUBB3*, *PPARγ*, *P53*, *CAT*, and *SOD2* mRNA Expression

**Fig. 5:**
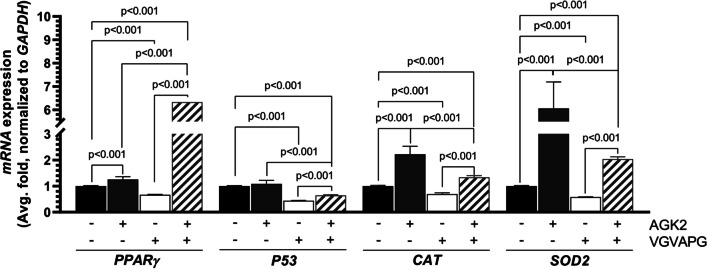
The tested hexapeptide induces changes in antioxidant stress-related genes. Results of *PPARγ*, *P53*, *CAT*, and *SOD2* mRNA expression after 6-h treatment of the SH-SY5Y-derived neurons with AGK2, VGVAPG, and VGVAPG/AGK2. The data are presented as means ± SD. The p values of one-way ANOVA, compared to the control, as well as p values shown by the t-test analysis between certain groups are denoted under the bars. The black, dark grey, white, and patterned bars show the results after the treatment with the vehicle (Control), VGVAPG, AGK2, and VGVAPG/AGK2, respectively

The *PPARγ* gene expression increased by 25.60% and 532.82% in the AGK2- and VGVAPG/AGK2-treated neurons, respectively, compared to the control (Fig. 5B). In turn, the VGVAPG treatment resulted in a 33.34% decrease in the *PPARγ* mRNA expression, compared to the control (Fig. 5B).

The *P53* gene expression decreased by 55.94% and 35.18% in the VGVAPG- and VGVAPG/AGK2-treated neurons, respectively, compared to the control (Fig. 5B).

The differentiated SH-SY5Y cells treated with AGK2 and VGVAPG/AGK2 were characterized by a 122.22% and 33.78% increase in the *CAT* mRNA expression, respectively, compared to the control (Fig. 5B). In turn, the *CAT* gene expression decreased significantly by 30.58% in the VGVAPG-treated cells (Fig. 5B).

The *SOD2* mRNA expression increased by 505.58% and 103.91% in the AGK2- and VGVAPG/AGK2-treated cells, respectively, compared to the control (Fig. 5B). In turn, a 41.83% decrease in the expression of this gene was observed in the VGVAPG alone-treated cells (Fig. 5B).

### *TUBB3*, *SIRT2* mRNA, and Protein Expression Level

**Fig. 6:**
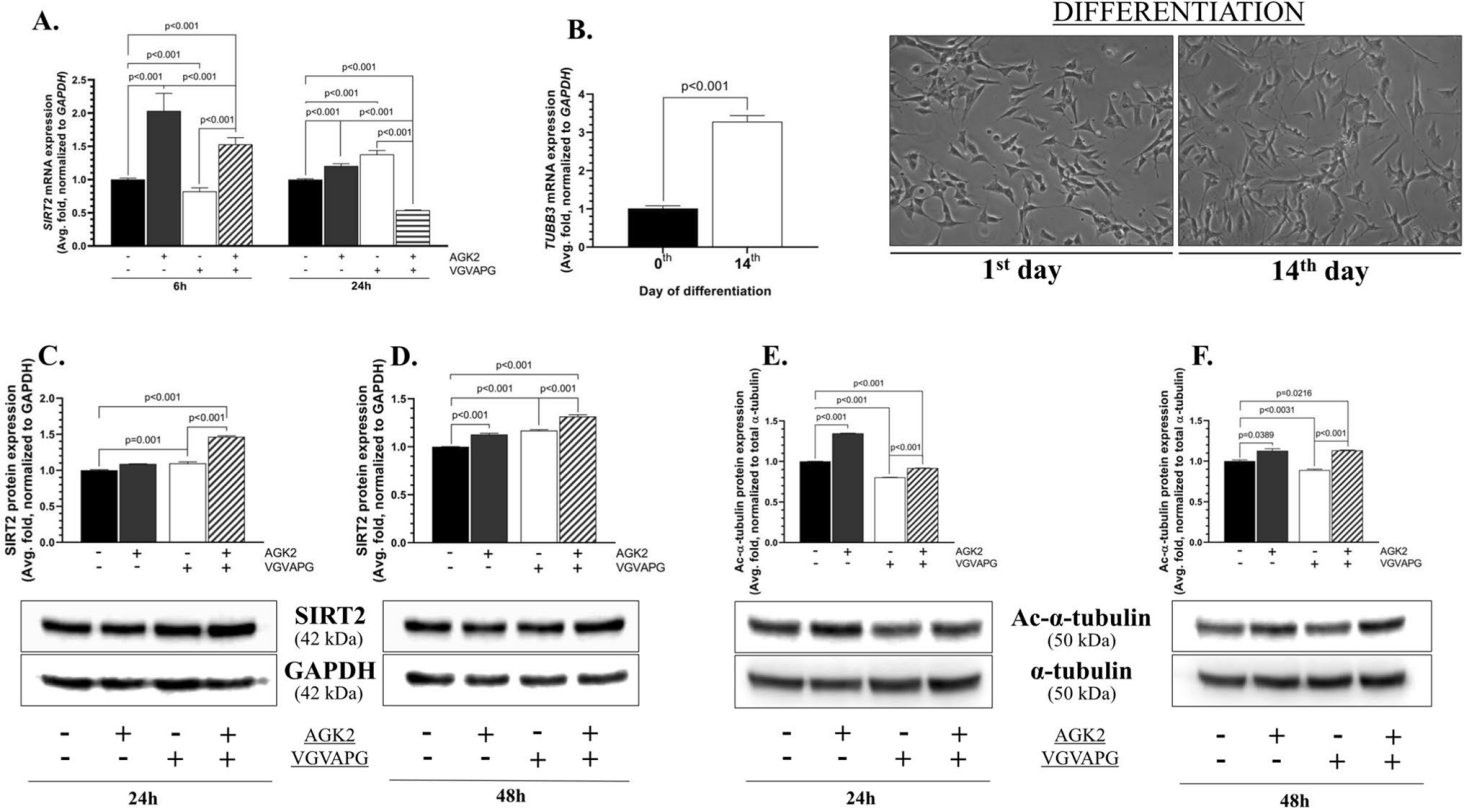
VGVAPG causes overexpression of SIRT2 at the protein and mRNA level and reduces the α-tubulin acetylation level. Results of mRNA expression of *SIRT2* (A) and *TUBB3* (B) and relative protein expression of Sirtuin 2 (C, D) and acetylated-α-tubulin (E, F) in the SH-SY5Y-derived neurons after treatment with AGK2, VGVAPG, and VGVAPG/AGK2 for 24 h and 48 h. Representative blots are shown below the graphs. Representative images captured on the 1^st^ and 14^th^ days are shown in the right panels. The data are presented as means ± SD. The p values of one-way ANOVA, compared to the control, as well as p values shown by the t-test analysis between certain groups are denoted under the bars. The black, dark grey, white, and patterned bars show the results after the treatment with the vehicle (Control), VGVAPG, AGK2, and VGVAPG/AGK2, respectively

After 6h, the *SIRT2* gene expression after the treatment with AGK2 and VGVAPG/AGK2 increased by 76.17% and 32.52%, respectively, compared to the control (Fig. 6A). In turn, the SH-SY5Y derived neurons exhibited a 29.99% decrease in the mRNA expression, compared to the control (Fig. 6A). Moreover, the effect differed between the VGVAPG- and VGVAPG/AGK2-treated cells (a 61.43% increase in the latter variant, compared to the VGVAPG alone treatment) (Fig. 6A).

After 24h, the *SIRT2* gene mRNA expression increased by 20.19% in the AGK2-treated cells, compared to the control (Fig. 6A). A similar effect was observed in the differentiated SH-SY5Y cells treated with VGVAPG, where a 37.82% increase in the expression of this gene was found, compared to the control (Fig. 6A). In turn, the *SIRT2* mRNA expression decreased by 46.34% in the VGVAPG/AGK2-treated cells, compared to the control (Fig. 6A). Moreover, a significant difference in the *SIRT2* gene expression was recirded between the VGVAPG- and VGVAPG/AGK2-treated cells (an 84.16% decrease in the latter variant, compared to the VGVAPG-treated cells) (Fig. 6A).

The *TUBB3* mRNA expression was 228.26% higher on the 14th day of differentiation than in the differentiation medium non-treated cells (Fig. 6B).

After 24h, the SIRT2 protein expression increased by 9.51% and 46.64% in the VGVAPG-treated and VGVAPG/AGK2-co-treated cells, respectively, compared to the control (Fig. 6C). A statistical decrease (by 37.13%) in the expression of this protein was observed in the VGVAPG-treated cells, compared to the VGVAPG/AGK2-co-treated cells (Fig. 6C). In turn, the SIRT2 protein expression after 48h significantly increased in all the tested variants and reached 12.82%, 16.78%, and 31.62% in the AGK2-, VGVAPG-, and VGVAPG/AGK2-treated cells, respectively, compared to the control (Fig. 6D). Moreover, the aforementioned increase in the SIRT2 protein expression in the VGVAPG-treated cells was 14.84% lower than in the VGVAPG/AGK2-co-treatment variant (Fig. 6D).

After 24h, the differentiated SH-SY5Y cells treated with AGK2 exhibited a 34.71% increase in the level of acetylated-α-tubulin (ac-α-tubulin), compared to the control (Fig. 6E). In turn, the protein expression decreased by 19.68% in the VGVAPG-treated cells, compared to the control (Fig. 6E). This effect was 11.44% lower in the VGVAPG-treated cells than in the VGVAPG/AGK2-co-treatment variant (Fig. 6E). After 48h, a 12.77% and 13.32% increase in the ac-α-tubulin level was observed in the AGK-2 and VGVAPG/AGK2-treated cells, respectively, compared to the control (Fig. 6F). In turn, a 10.95% decrease in the expression of this protein was recorded in the VGVAPG-treated cells, compared to the control; moreover, this effect was weaker by 24.27% than in the VGVAPG/AGK2-co-treated cells (Fig. 6F).

## Discussion

Differentiated SH-SY5Y cells are one of the common models of mature neurons used in screening studies, which is associated with their expression of molecular markers and tau protein sorting mechanism typical of mature neurons [19]. Usually, the *TUBB3* mRNA expression is measured to prove the efficiency of the differentiation process [32]. Our results show over threefold higher *TUBB3* gene expression in the SH-SY5Y cells on the 14^th^ day of differentiation, compared to the non-differentiated cells. Similarly, their morphology after the differentiation process was characteristic of neuronal cells, i.e. elongated and extensive neurites. These findings prove a successful differentiation process and are in agreement with the results reported *inter alia* by Tong et al. or Martin et al. [33, 34].

Firstly, we decided to carry out preliminary determination of the impact of VGVAPG on basic neuronal cell parameters. The metabolic activity (resazurin reduction assay) and intracellular ROS level (H_2_DCF-DA probe) methods were chosen to assess if there is any correlation between the VGVAPG mechanism of action and SIRT2 (using AGK2 – a potent SIRT2 inhibitor). The results of the resazurin reduction assay showed that VGVAPG per se did not significantly affect the cell metabolic activity after the 24-h and 48-h treatment, while a significant decrease in the intracellular ROS level was observed. On the other hand, the pretreatment of the neurons with AGK2 and the subsequent treatment with VGVAPG caused a significant decrease in the intracellular ROS level. Moreover, after 48h, this effect was statistically different from that in the VGVAPG alone-treated cells. These results are in line with previous studies on undifferentiated SH-SY5Y cells, human lung adenocarcinoma (A549), breast cancer cells (MCF-7), and murine embryo skin fibroblasts (3T3-L1) reported by our team [9, 35, 36]. The results obtained in this study show some impact of the tested hexapeptide on SIRT2 as well as the ability of VGVAPG to dysregulate the intracellular ROS metabolism in the neurons. Such outcomes are important due to the well-described ability of free radicals to induce apoptosis [37].

Although the tested hexapeptide did not show the ability to affect the ROS level, we decided to determine its impact on CAT and SOD activity due to their indicative role in the occurrence of oxidative stress in cells. Our results demonstrated that the tested hexapeptide alone did not affect CAT activity, in contrast to AGK2, which caused a decrease in this parameter in both time intervals. These findings are in line with the results of ROS level measurements, which did not show an increase in this parameter. Szychowski et al. have also shown that VGVAPG can decrease the CAT protein level in undifferentiated SH-SY5Y cells [9]. No other studies of the impact of VGVAPG on CAT activity, mRNA, and/or protein expression are available. In turn, the activity of SOD did not show any significant changes after 24h but decreased in the 48-h treatment variant. This finding is in contrast to the results shown in a previous study, which reported an increase in SOD1 protein expression in mouse astrocytes treated in vitro with the same concentration of VGVAPG [38]. However, the authors used a non-human-origin cell model; moreover, it is well-known that the role of astrocytes in the brain is quite different from that played by neurons, which may explain the different findings. On the other hand, the *SOD2* gene is regulated by the FOXO3 transcriptional factor, which first needs to be deacetylated by SIRT2 [39]. The massive decrease in the SOD activity may be therefore correlated with some effect of VGVAPG on SIRT2 activity. Nevertheless, the results presented above do not show the ability of VGVAPG to induce oxidative stress in differentiated neuronal SH-SY5Y cells. However, to fully elucidate the impact of VGVAPG on neuronal cells, we decided to determine whether the tested hexapeptide shows pro-apoptotic properties.

We chose the activity of caspase-3 as a parameter indicating potential pro-apoptotic properties of VGVAPG, based on the engagement of caspase-3 in the active phase of apoptosis. The present results show that the inhibition of SIRT2 via AGK2 significantly increased caspase-3 activity, in contrast to the VGVAPG peptide applied alone. Interestingly, the pre-treatment of the neuronal cells with AGK2 and the subsequent treatment with the tested hexapeptide enhanced this effect. A similar cumulative effect on this parameter was observed by Wawruszak et al. in breast cancer luminal cells treated with paclitaxel and AGK2 [40]. This may prove the above-mentioned interaction between the tested peptide and SIRT2. In turn, Wang et al. have shown the ability of AGK2 to decrease cleaved caspase-3 expression in murine brain [41]. Interestingly, the results obtained in the next step of our study showed a similar ability of VGVAPG to reduce the axon length to that of AGK2. The loss of synapses, axon fragmentation, and/or dysmorphism are the most characteristic features of neurodegeneration [42]. As demonstrated by Jiao et al., treatment of BV2 microglial cells with AGK2 caused degenerative morphological changes in these cells, likewise in our study [43]. However, only one parameter of neuronal cell morphology was determined in our study, which needs to be extended in the future. Nevertheless, the shortening of the neurite length, similar to the AGK2 effect, may indicate disruption of the cytoskeleton regulation in the neuronal cell model; therefore, we further decided to determine the impact of VGVAPG on the acetylation level of α-tubulin, which is one of the substrates of NAD + -dependent activity of SIRT2.

Since there were no significant changes in the above-described parameters, we decided to check the impact of VGVAPG on the expression of inflammation- (*PPARγ)*, apoptosis- (*P53)*, and oxidative stress- (*CAT,* *SOD*)-related genes. The downregulation of the expression of these genes suggests a disruptive role of the tested hexapeptide on certain intracellular pathways in differentiated SH-SY5Y cells. The decrease in the *CAT* and *SOD2* mRNA expression is in line with the results shown in this study, which prove the ROS-independent mechanism of the VGVAPG action. As reported by Okuno et al. and Kim, the regulation of *CAT* and *SOD* mRNA expression is proportional to PPARγ activity [44, 45]. Some reports have shown that certain sirtuins interact with PPARγ, e.g. Han et al. have demonstrated that Sirtuin 1 is negatively controlled by this receptor during adipogenesis [46]. However, the activity of this receptor is commonly linked with the anti-degenerative and anti-inflammation effect, which was presented *inter alia* by Inestrosa et al. in rat hippocampal neurons (abolishment of the Aβ effect after Pparγ activation) or increased neuronal death in Pparγ-knock-out mouse primary neurons after ischemic stroke, which was also correlated with a decrease in CAT and SOD protein expression [47]. PPARγ also controls the *P53* gene expression by binding to its promoter [48]. This explains the decrease in the expression of the *P53* gene encoding the p53 protein, which was proportional to the *PPARγ* mRNA expression induced by VGVAPG. Indeed, in their review, Polvani et al. have shown that PPARγ acts as a transcriptional factor regulating the expression of certain genes engaged in the cell antioxidant system and apoptosis [49]. Therefore, we suppose that VGVAPG induces neurodegeneration, which is a trigger factor for *PPARγ* expression; however, little is known about SIRT2-PPARγ interactions in neuronal cell models, while SIRT2 activity is necessary for maintenance of *redox* homeostasis in neurons and stabilization of the cytoskeleton. Therefore, in the next step of the study, we decided to assess the potential effect of VGVAPG on SIRT2 and its activity.

SIRT2 is a NAD^+^-dependent deacetylase with a confusing role in certain tissues. It has been shown that blocking of its activity with certain inhibitors in some cells exerts positive and negative effects [15, 22, 51]. Our results are the first to show the decrease in the *SIRT2* mRNA expression after 6h of treatment with VGVAPG, in contrast to the 24-h exposure of the differentiated SH-SY5Y cells to VGVAPG, which exhibited a significant increase in the expression of this gene. Furthermore, the increase in the SIRT2 protein expression was observed in both tested time intervals (24h and 48h) in the VGVAPG-treated neurons. This indicates that there is some correlation between VGVAPG and SIRT2, which may be engaged in neurodegeneration induction. Indeed, Sidorova-Darmos et al. have found that the increase in the *Sirt2* mRNA and protein expression in rat brains during aging may be correlated with neurodegeneration [18]. Moreover, many papers highlight the importance of SIRT2 in neurodegenerative diseases, *inter alia* in AD [15]. In turn, Singh et al. have shown that SIRT2 protein overexpression may be correlated with defense against oxidative stress (induced by chemical compounds) in SH-SY5Y cells [51]. Similar results were reported by You et al., who proved the correlation between changes in SIRT2 expression and oxidative stress (caused by high-glucose uptake) [52]. However, the present results did not indicate induction of oxidative stress in the differentiated SH-SY5Y neurons by VGVAPG. Therefore, it can be assumed that the tested hexapeptide may have a different mechanism of action, but its ability to affect the SIRT2 protein and mRNA expression is undisputed.

According to the literature, SIRT2 is responsible for stabilization of the cytoskeleton by ensuring the homeostasis of the tubulin acetylation level. It is well-known that the acetylation of tubulin monomers is connected with their stability and self-regeneration processes. In contrast, a decrease in acetylation is mainly correlated with neurodegeneration [53–55]. Our data show that VGVAPG causes a massive decrease in the ac-α-tubulin level after 24-h and 48-h treatment, which is strictly correlated with an increase in the level of SIRT2 protein expression. Therefore, it may be supposed that the tested hexapeptide is involved in the degeneration of neurites through induction of SIRT2 overexpression, which subsequently leads to a decrease in tubulin acetylation. Recently, Saunders et al. have proved that acetylation of α-tubulin is crucial for the stability of this protein in *Drosophila melanogaster* as well as neuron growth and synapse morphogenesis [56]. Moreover, Wei et al. showed that blocking of α-tubulin acetylation in MEK-17 mice neurons caused their overbranching and overgrowth [57]. Furthermore, the deficiency in the acetylation level has been directly linked with AD *inter alia* by Zhang et al., who detected a decreased level of ac-α-tubulin in the brains of AD patients and in an aged-mice in vivo model [58]. On the other hand, Fourcade et al. have shown that *Sirt2* knock-out mice are characterized by degeneration of axons, which leads to locomotor disfunctions in such animals, proving the role of this deacetylase in the maintenance of proper neuronal function [59]. Moreover, earlier studies (2007 and 2010) have demonstrated a positive effect of SIRT2 (genetic or pharmacological) inhibition in Huntington’s disease (HD) and Wallerian degeneration [60, 61]. However, both cited authors used different models of different neurodegenerative diseases, which may be the cause of the contrasting results. Considering the above-cited papers and the present results, we assume that the tested hexapeptide stimulates *PPARγ* mRNA expression via a ROS-independent, degeneration-dependent pathway together with overexpression of SIRT2, which subsequently causes a decrease in the acetylation level of cytoskeleton elements, resulting in the degeneration of neuronal cells.

## Conclusions

The present study shows for the first time that the elastin-derived VGVAPG peptide is able to cause neurodegeneration reflected in the shortening of the length of axons. Moreover, the ability of this hexapeptide to induce fluctuations in *SIRT2* mRNA and an increase in the expression of this protein has been evidenced, which we suppose is the main factor of the destabilization of the cytoskeleton, triggering the *PPARγ* mRNA expression. Based on the obtained results, the ROS-dependent mechanism of action of VGVAPG in SH-SY5Y neuronal cells in vitro has been excluded.

## Supplementary Information

Below is the link to the electronic supplementary material.

Supplementary file1 (PDF 186 KB)

## Data Availability

Data is available on reasonable request.

## Abbreviations

AD: Alzheimer’s disease
AGK2: 2-Cyano-3-[5-(2,5-dichlorophenyl)-2-furanyl]-N-(5-quinolinyl)-2-propenamide
BSA: Bovine serum albumin
EDPs: Elastin-derived peptides
ELPs: Elastin-like polypeptides
FBS HI: Fetal bovine serum heat inactivated
H_2_DCF-DA: 2',7'-Dichlorodihydrofluorescein diacetate
NAD^+^: Oxidized nicotinamide adenine dinucleotide
ROS: Reactive oxygen species
SIRT2: Sirtuin 2
VGVAPG: Val-Gly-Val-Ala-Pro-Gly
