# Supplementary material for: Interaction Between Aging-Related Elastin-Derived Peptide (VGVAPG) and Sirtuin 2 and its Impact on Functions of Human Neuron Cells in an In Vitro Model
Source: Mol Neurobiol. 2024 Jun 24;62(1):819–31. doi: 10.1007/s12035-024-04298-y (PMC11711152; doi:10.1007/s12035-024-04298-y)
Supplement: Supplementary file 1 — Supplementary file1 (PDF 186 KB) [file 12035_2024_4298_MOESM1_ESM.pdf]

Raw blots

SIRT2 – 24h and 48h – mAb (cat. A3967)

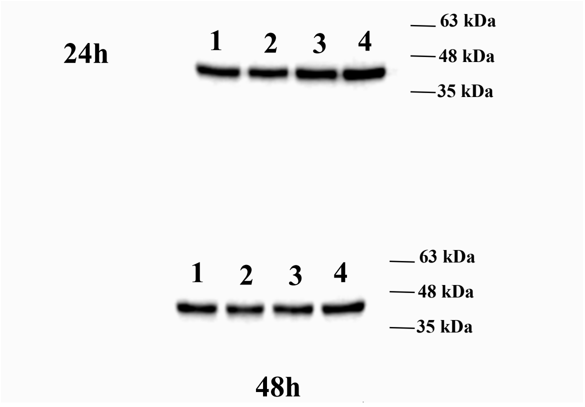

1 – Control (DMSO)
2 – 10 μM AGK2
3 – 10 nM VGVAPG
4 - 10 nM VGVAPG/10 μM AGK2

GAPDH – 24h and 48h – mAb (cat. sc-47724)

1 – Control (DMSO)
2 – 10 μM AGK2
3 – 10 nM VGVAPG
4 - 10 nM VGVAPG/10 μM AGK2

24h

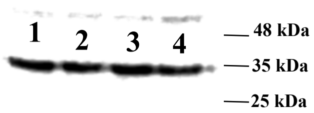

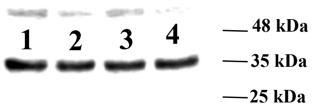

48h

Ac-α-tubulin – 24h and 48h – mAb (cat. sc-23950)

1 – Control (DMSO)
2 – 10 μM AGK2
3 – 10 nM VGVAPG
4 - 10 nM VGVAPG/10 μM AGK2

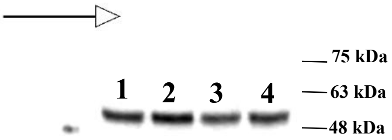

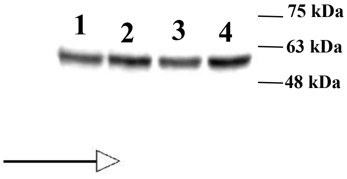

A-tubulin – 24h and 48h – mAb (cat. 66031-1-Ig)

48h

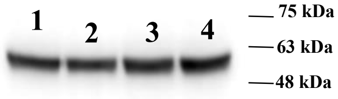

1 – Control (DMSO)
2 – 10 μM AGK2
3 – 10 nM VGVAPG
4 - 10 nM VGVAPG/10 μM AGK2

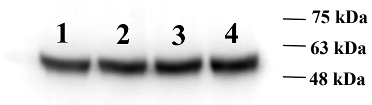

24h
